# Folic Acid Inhibits Amyloid β-Peptide Production through Modulating DNA Methyltransferase Activity in N2a-APP Cells

**DOI:** 10.3390/ijms161025002

**Published:** 2015-10-20

**Authors:** Wen Li, Mingyue Jiang, Shijing Zhao, Huan Liu, Xumei Zhang, John X. Wilson, Guowei Huang

**Affiliations:** 1 Department of Nutrition and Food Science, School of Public Health, Tianjin Medical University, 22 Qixiangtai Road, Heping District, Tianjin 300070, China; E-Mails: liwen08648@126.com (W.L.); jiang2011602151@126.com (M.J.); zhao2011602144@126.com (S.Z.); liuhuan80@126.com (H.L.); zhangxumei2013@126.com (X.Z.); 2 Department of Exercise and Nutrition Sciences, School of Public Health and Health Professions, University at Buffalo, Buffalo, NY 14214, USA; E-Mail: jxwilson@buffalo.edu

**Keywords:** Alzheimer’s disease, folic acid, DNA methyltransferases, amyloid β-peptide

## Abstract

Alzheimer’s disease (AD) is a common neurodegenerative disease resulting in progressive dementia, and is a principal cause of dementia among older adults. Folate acts through one-carbon metabolism to support the methylation of multiple substrates. We hypothesized that folic acid supplementation modulates DNA methyltransferase (DNMT) activity and may alter amyloid β-peptide (Aβ) production in AD. Mouse Neuro-2a cells expressing human APP695 were incubated with folic acid (2.8–40 μmol/L), and with or without zebularine (the DNMT inhibitor). DNMT activity, cell viability, Aβ and DNMTs expression were then examined. The results showed that folic acid stimulated *DNMT* gene and protein expression, and DNMT activity. Furthermore, folic acid decreased Aβ protein production, whereas inhibition of DNMT activity by zebularine increased Aβ production. The results indicate that folic acid induces methylation potential-dependent DNMT enzymes, thereby attenuating Aβ production.

## 1. Introduction

Alzheimer’s disease (AD) is one of the major amyloidoses, with two types of amyloid deposited in the brain: (i) amyloid β-peptide (Aβ) forming aggregates senile plaques and cerebrovascular amyloid angiopathy [1]; and (ii) tau protein, which forms neurofibrillary tangles, neuropil threads, and dystrophic neurites [2]. There is no cure for AD currently [3]. However, malnutrition is a potential risk factor for cognitive impairment [4], there is a strong rationale to search for nutritional interventions for AD and to understand their molecular mechanisms of action.

The epigenetic mechanism of gene methylation provides a putative link between nutrition, one-carbon metabolism and disease progression, because dietary deficiency in transmethylation micronutrients (e.g., folate) may cause hypomethylation of promoter regions in AD-relevant genes. This effect is observed in AD models, for example in TgCRND8 mice which carrying mutant amyloid precursor protein (APP), fed a diet deficient in three kinds of vitamin B (folate, vitamin B12 and B6) [5]. The diet inhibits the metabolism of homocysteine by transsulfuration and remethylation pathways in TgCRND8 mice [6]. The resulting hyperhomocysteinemia is associated with a demethylation of the *PS1* gene promoter that is not attributable to the deposition of Aβ in neuritic plaques.

Instead the gene demethylation may be due, at least partially, to depletion of *S*-adenosylmethionine (SAM) and elevation of intracellular *S*-adenosylhomocysteine (SAH), resulting in an overall decrease of methylation potential. In particular, SAH concentration rises to levels that competitively inhibit the SAM-dependent methylation of genes by DNA methyltransferases (DNMT) [6,7]. Hyperhomocysteinemia and DNA hypomethylation are associated with elevated PS1 expression and Aβ production [7]. In neuroblastoma cell cultures, folate/B6/B12-deprivation leads to accumulation of homocysteine, diminution of the methylation potential, and upregulation of PS1 and *A*β gene expression, whereas SAM administration downregulates *PS1* gene expression and Aβ production [8,9].

The folate/B6/B12-dependent mechanisms described above may occur in clinical AD [10]. Because folic acid supplementation is a potential therapeutic intervention, the study of the effects of exogenous folate independently of B6 and B12 is important. In this study we hypothesized that folic acid supplementation may alter Aβ production in N2a-APP cells because the DNMT expression and activity can be modulated.

## 2. Results

### 2.1. Amyloid β-Peptide Protein

**Figure 1:**
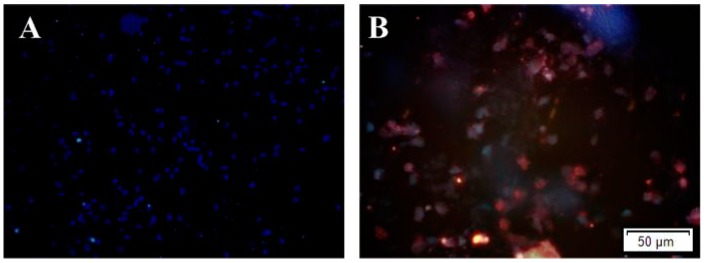
The comparison of Aβ production levels between N2a-WT and N2a-APP cells. Both WT-N2a and N2a-APP cells were cultured with 10 μmol/L folic acid. Subsequently Aβ was detected by immunofluorescence staining (red), nucleus staining by DAPI (blue). (**A**) Representative images showing Aβ immunofluorescence in N2a-WT cells; (**B**) Representative images showing Aβ immunofluorescence in N2a-APP cells. Scale bar = 50 μm.

**Figure 2:**
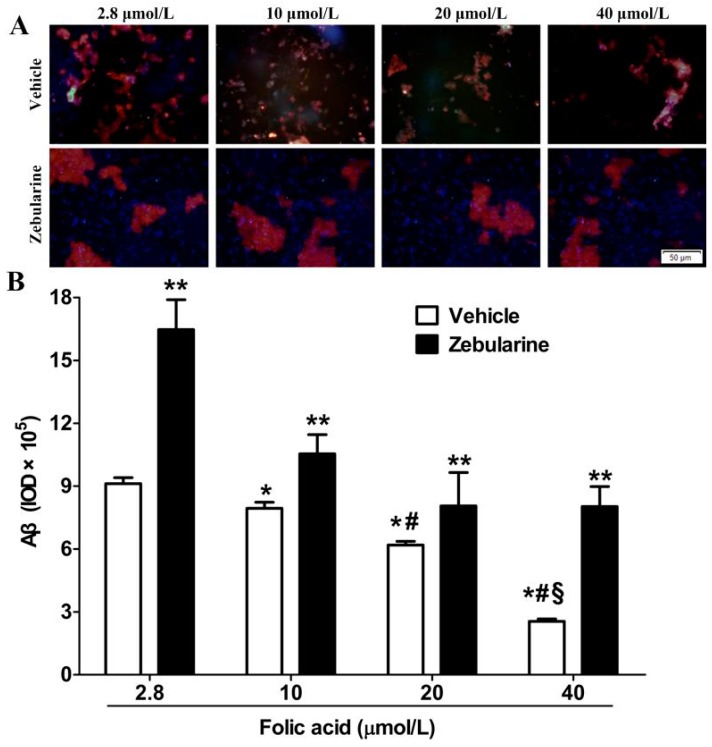
Folic acid decreases, and zebularine increases, Aβ production in N2a-APP cells. The cells were incubated with 2.8, 10, 20 or 40 μmol/L folic acid for 96 h, and with either zebularine or its vehicle present during the first 12 h. Subsequently Aβ was detected by immunofluorescence staining (red), nucleus staining by DAPI (blue). (**A**) Representative images showing Aβ immunofluorescence; (**B**) Summary of Aβ production levels shows mean ± SEM for integrated optical density (IOD) of immunoreactive Aβ in 3 experiments. * *p* < 0.05 compared with 2.8 μmol/L. # *p* < 0.05 compared with 10 μmol/L. § *p* < 0.05 compared with 20 μmol/L. ** *p* < 0.05 compared with vehicle at the same folate concentration. Scale bar = 50 μm.

Almost no expression of Aβ production was observed in N2a-WT cells. High expression of Aβ production due to the stable expression of human APP695 was seen in N2a-APP cells (Figure 1). Immunofluorescence staining of N2a-APP cell cultures revealed that folic acid (2.8 to 40 μmol/L) caused a dose-dependent decrease in immunoreactive Aβ (Figure 2). Incubation of the cells with zebularine increased Aβ immunoreactivity at each folic acid concentration (Figure 2).

### 2.2. DNMT Activity

**Figure 3:**
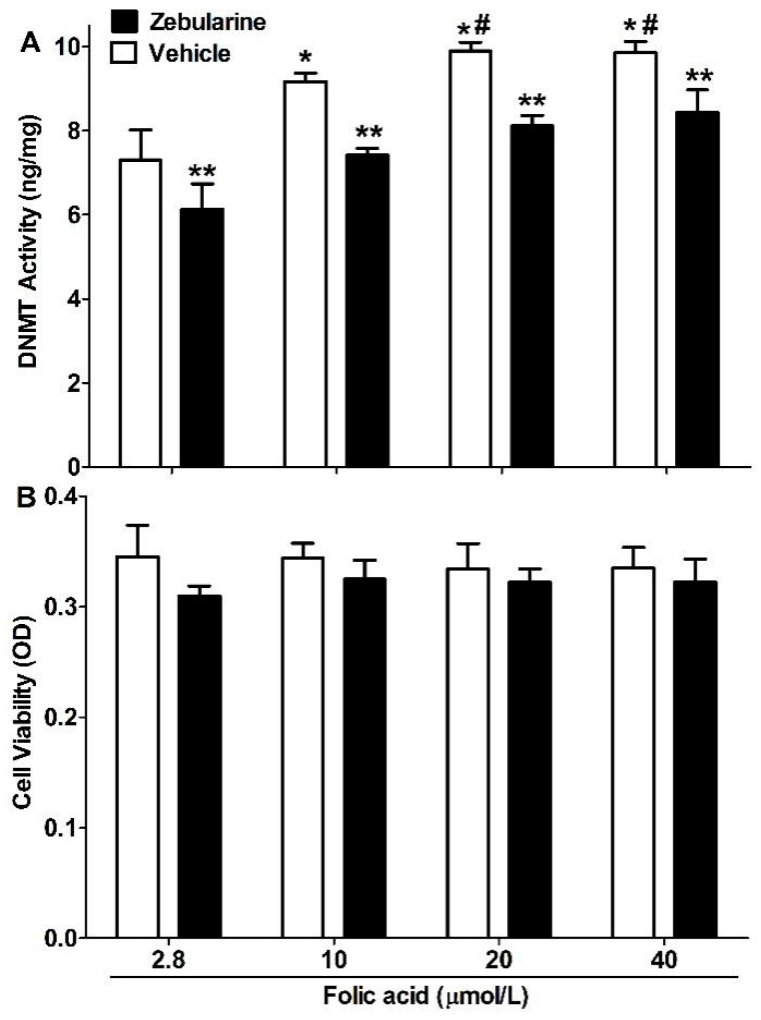
Folic acid raises and zebularine lowers DNMT activity, without altering cell viability. N2a-APP cells were incubated as described in Figure 1. (**A**) DNMT activity in nuclear extracts; (**B**) Cell viability based on Alamar blue assay. The plotted values are mean ± SEM values for 3 experiments. * *p* < 0.05 compared with 2.8 μmol/L. # *p* < 0.05 compared with 10 μmol/L. ** *p* < 0.05 compared with vehicle at the same folic acid concentration.

DNMT activity in N2a-APP cells increased with folic acid concentration (2.8 to 20 μmol/L) and was partially inhibited by zebularine (Figure 3A). Neither folic acid nor zebularine altered cell viability (Figure 3B).

### 2.3. DNMT Expression

**Figure 4:**
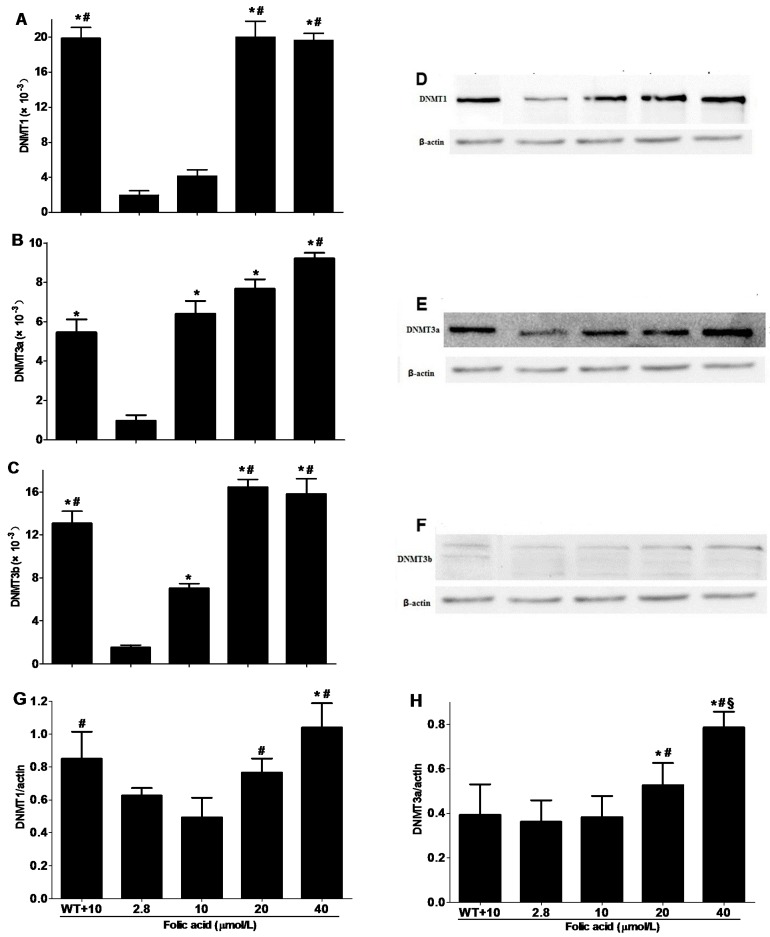

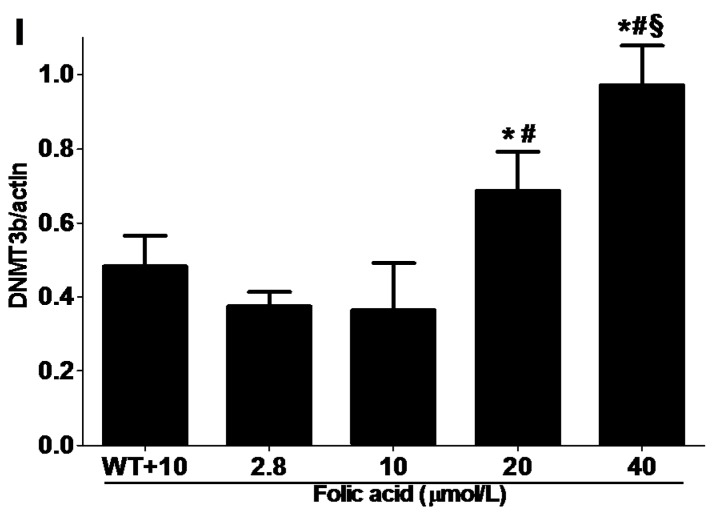
Folic acid increases the expression of DNMT isoforms in N2a cells. Wild type N2a cells were incubated with 10 μmol/L folic acid (WT + 10) and N2a-APP cells were incubated with 2.8–40 μmol/L folic acid for 96 h. (**A**–**C**) Gene expression levels of *DNMT1*, *DNMT3a* and *DNMT3b* in N2a and N2a-APP cells (*n* = 3 experiments); (**D**–**F**) Representative western blots of DNMT isoforms and actin in N2a and N2a-APP cells; (**G**–**I**) Summaries of densitometric analyses of western blots of DNMT isoforms and actin in N2a and N2a-APP cells (*n* = 3 experiments). * *p* < 0.05 compared with 2.8 μmol/L; # *p* < 0.05 compared with 10 μmol/L; § *p* < 0.05 compared with 20 μmol/L.

The levels of gene expression of DNMT1 and DNMT3b were lower in N2a-APP cells than wild type N2a cells when the culture medium contained folic acid 10 μmol/L (Figure 4A,C). However, *DNMT3a* gene expression did not differ between the cell types (Figure 4B). Gene expression of all the DNMT isoforms increased with folic acid concentration (2.8 to 20 μmol/L) in N2a-APP cells (Figure 4A–C).

The protein expression levels of DNMT1 were lower in N2a-APP cells than wild type N2a cells when the culture medium contained 10 μmol/L. However, DNMT3a and DNMT3b protein expression did not differ between the cell types. Folic acid caused a dose-dependent increased protein expression of all the DNMTs in N2a-APP cells (Figure 4D–I).

## 3. Discussion

The present study found that folic acid dose-dependently inhibited Aβ production in N2a-APP cells. Further observations indicated that this effect of folic acid was mediated by stimulated *DNMT* gene and protein expression, and DNMT activity. Furthermore, folic acid decreased Aβ protein production, whereas inhibition of DNMT activity by zebularine increased Aβ production. Taken together, these results indicate that folic acid induces methylation potential-dependent DNMT enzymes attenuating Aβ production.

Genetic factors have an important role in the development of late-onset AD (LOAD). AD, especially LOAD, arises mainly from heritable causes. Yet, the etiology of AD is complex and non-Mendelian, so the epigenetic mechanism in AD development should also be noted [11]. DNMT3A and DNMT3B establish DNA methylation patterns, and DNMT1 subsequently maintains it. The general characteristic of age-related diseases and aging are hypomethylation of genome and hypermethylation of specific genes promoter. Aberrant epigenetic control in CpG-island may contribute to late-onset AD pathology [12]. Some studies showed that some genes which participate in methylation homeostasis (e.g., DNMT1, MTHFR) and Aβ processing (e.g., APOE, PSEN1) show a significant epigenetic variability, those genes may contribute to late-onset AD predisposition. Mastroeni’s study detected neuronal immunoreactivity of epigenetic factors, including DNMT1, in AD cases whose epigenetic factors were significant decrements in AD-vulnerable neurons [13]. Thus, the expression of DNMT1 was decreased in the AD cell model (N2a-APP cells) as compared with the wild type cells for which culture medium contains 10 μmol/L folic acid.

The stimulation by folic acid attenuating Aβ production could be explained by the increases in DNMT activity that was observed in N2a-APP cells. The rise in DNMT activity supported DNA methylation modulated by DNMT isoforms. Also consistent with this explanation were the effects of zebularine, which is a validated inhibitor of DNMT [14,15].

In addition folic acid elevated the gene expression and protein levels of DNMT1, DNMT3a and DNMT3b in N2a-APP cells. Folic acid evidently induced expression of functional DNMT isoforms, because it increased the DNMT activity measured in nuclear extracts. An essential role of DNMT activity in protecting against experimental AD progression was revealed by the observation that the DNMT inhibitor zebularine stimulated Aβ production at each folic acid concentration in N2a-APP cell cultures.

Recent studies revealed that experience factors could mediate DNA methylation, which is regulated by DNMT activity, and DNMT activity is related to recent memory formation and as well as remote memory maintenance [16,17]. Notably, the expression of DNMT3a was detected in the hippocampus in aged mice, DNMT3a overexpression can reverse the deficit especially in spatial memory [18,19]. However, a decline in global DNA methylation was found in the autopsied hippocampus of patients with AD [20,21].Moreover, Guo’s study indicated that histone H3 hyperacetylation and DNMT-dependent hypomethylation mediate the stress-related signaling pathways activation in SH-SY5Y cells, which lead to *APP*, *PSEN1*, and *BACE1* genes expression increase, thus leading to Aβ overproduction [22]. Thus, we speculate that folic acid modulates the activity of DNMT to ameliorate Aβ production.

A randomized controlled trial in elderly patients with mild to moderate AD found that cognitive decline did not slow by supplements of folic acid, B6 and B12 during the 18-month follow-up [23]. It should be noted that the trial excluded individuals if they had subnormal serum levels of folate or vitamin B12 [24], although a review of other studies found that serum folate concentration typically is lower in AD patients than controls [25]. However, in contrast to studies of cognitive decline, the VITACOG randomized controlled trial used structural neuroimaging of the brain to assess the effect of supplementation with folic acid, B6 and B12 in elderly patients with mild cognitive impairment [26]. The VITACOG trial found that this supplementation decreased atrophy in brain regions specifically vulnerable to the AD process [26].

## 4. Experimental Section

### 4.1. In Vitro Model

The experimental protocols of cells were approved by Tianjin Medical University Animal Ethics Committee (Study number: TMUaMEC 2012016; Tianjin, China). Mouse neuroblastoma N2a cells stably expressing human APP695 (N2a-APP cells) and wild type N2a cells were obtained from Huaxi Xu (Biomedical Research, Xiamen University, Xiamen, China) [27]. The N2a-APP cells were maintained in Dulbecco’s Modified Eagle’s Medium (DMEM)/Opti-MEM (1:1, *v*/*v*), with 200 μg/mL G418, 10% fetal bovine serum, 100 mg/mL streptomycin, 100 units/mL penicillin, at 37 °C in 5% CO_2_/95% air. Both cell types were passaged every 3 days when growing to 80% confluence. Folic acid-free DMEM powder was purchased from Gibco-BRL (Paisley, UK) and combined with predetermined amounts of folic acid to make culture media for the experiment. The N2a-APP cells were exposed to folic acid (2.8–40 μmol/L) for the 96 h. Either the DNMT inhibitor zebularine (30 μmol/L) or its vehicle was added for the first 12 h, the medium was changed after first 12 h to remove zebularine or its vehicle from the cell cultures.

### 4.2. Immunofluorescence Staining

Aβ protein in N2a-APP cell cultures was detected by immunofluorescence staining. Cell cultures on laminin-coated coverslips were fixed 20 min in 4% paraformaldehyde at room temperature, washed with PBS, and then blocked 1 h with 10% goat serum in PBS. Slides were incubated at 4 °C over night with the primary antibody (anti-Aβ (to detect Aβ 1–42 and Aβ 1–40), 1:200; Sigma, St. Louis, MO, USA) [28]. After repeated PBS rinses of slides, the secondary antibody (1:100; Jackson ImmunoResearch Laboratories; West Grove, PA, USA) was applied at room temperature for 2 h. PBS was used to wash and incubate slides. Ten images were obtained from each slide by indirect fluorescence using a fluorescent microscope X81 (Olympus, Tokyo, Japan) and the integrated optical density of each was determined with Image-Pro Plus 6.0 software (Media Cybrnetics, Silver Spring, MD, USA).

### 4.3. DNMT Activity

A nuclear extraction kit of Merck (Merck KGaA, Darmstadt, Germany) was used to isolate nuclear extracts of cells. These extracts was used to measure DNMT activity using the DNA Methyltransferase Activity/Inhibition Assay Kit of Active Motif (Carlsbad, CA, USA) [14]. According to the manufacturer’s instructions, DNMT1 provided in the kit was used to create a lot-specific standard curve. The microplate reader was used to measure optical density (OD) at 450 nm and DNMT activity (unit by OD/(h·mg)) was calculated using the following formula:

(1) $$\text{DNMT activity[OD/(h mg)]}=\frac{\left(\text{Average}\;\text{Sample}\;\text{OD}-\text{Average}\;\text{Blank}\;\text{OD}\right)}{\text{Protein}\;\text{amount}\;(\text{μg})*\times \text{hour}**}\times 1000$$

* Protein added for the reaction; ** Incubation time for the reaction.

### 4.4. Cell Viability

Alamar Blue (resazurin) assay was used to measure cell viability. Cells were incubated for 3 h with 0.1 mg/mL Alamar blue reagent (Gibco-BRL, Paisley, UK) in cell culture medium, then the optical density (OD) at 575 nm was read used microplate reader (Bio-Tek ELX800uv, Bio-Tek Instrument Inc., Winooski, VT, USA).

### 4.5. Real-Time PCR

Real-time PCR was used to quantify the genes expression. Trizol was used to extract total RNA of the cells according to the instructions of the manufacturer. First-strand cDNA was synthesized from total RNA (2 μg) using MMLV reverse transcriptase. The reaction mixture (20 μL) was incubated for 60 min at 42 °C, 10 min at 70 °C, and held at −20 °C. LightCycler 480 SYBR Green I Master Kit (Roche, Mannheim, Germany) was used to perform real-time PCR. The 20 μL PCR mixture included PCR Master (10 μL), cDNA (5 μL), forward primer (1 μL), reverse primer (1 μL) and water (PCR-grade) (3 μL). Those reaction mixtures were incubated 5 min at 95 °C, followed by 45 amplification cycles (denaturation, 95 °C for 10 s; annealing, 56 °C for DNMT3b, 59 °C for DNMT1 and DNMT3a for 10 s; extension, 72 °C for 10 s). Primers were specific for DNMT1 (forward, CTAGTTCCGTGGCTACGAG GAGAA; reverse, TCTCTCTCCTCTGCAGCCGACTCA), DNMT3a (forward, GCCGAATTGTGTCTTGGTGGATGACA; reverse, CCTGGTGGAATGCACTGCAGAAGGA), or DNMT3b (forward, TTCAGTGACCAGTCCTC AGACACGAA; reverse, TCAGAAGGCTGGAGACCTCCCTCTT). For each gene the expression was normalized to β-actin (forward, AATGTGTCCGTCGTGGATCT; reverse, GGTCCTCAGTGTAGCCCAAG) in order to calculate relative levels of transcripts.

### 4.6. Western Blot Analysis

Western blot analysis was used to assess protein expression of DNMT isoforms. Ice-cold PBS washed cells were lysed used TNE-NP40 buffer. Lysates of cells were separated by electrophoresis on 12% sodium dodecyl sulfate polyacrylamide gel then transferred to PVDF membranes. After blocking (5% non-fat milk),the PVDF membranes were incubated with primary antibodies (anti-DNMT1, 1:1000; anti-DNMT3a, 1:1000; anti-DNMT3b, 1:1000; anti-β-actin, 1:5000) overnight at 4 °C, antibody of DNMT1 and DNMT3a purchase from CST and antibody of DNMT3b and β-actin purchase from Abcam, after washed with PBST, incubated with appropriate secondary antibodies (IgG-horseradish peroxidase, Zhongshan Goldbridge Biotechnology, Beijing, China) at room temperature for l h. Chemiluminescence assay was used to detected the expression of proteins and then using NIH Image software 1.61 (Macintosh, CA, USA) quantified intensity, and normalized to β-actin band respectively.

### 4.7. Statistical Analysis

The plotted values are mean ± SEM values for 3 experiments. We used the statistical software package SPSS 16.0 to evaluate differences between groups used one-way ANOVA and either Tukey’s HSD test or Dunnet’s test. *p* < 0.05 was considered significant.

## 5. Conclusions

In conclusion, the present study using an AD cell model found that folic acid stimulated gene and protein expression of DNMT isoforms, and DNMT activity. Folic acid also decreased Aβ protein levels, whereas inhibition of DNMT activity by zebularine increased Aβ production. These results indicate that folic acid’s induction of methylation potential-dependent DNMT enzymes could consequently slow Aβ production. This novel finding may stimulate reinvestigation of folic acid supplementation as a treatment for AD patients.

## Abbreviations

AD: Alzheimer’s disease; Aβ: amyloid β-peptide; APP: amyloid precursor protein; PSEN: presenilin; N2a-APP: N2a neuroblastoma cells overexpressing APP695; DMEM: Dulbecco’s Modified Eagle’s Medium; SAH: *S*-adenosylhomocysteine; DNMT: DNA methyltransferase; SAM: *S*-adenosylmethionine; IOD: integrated optical density.
